# Using Serum Lactate Concentration to Correct for the Anion Gap

**DOI:** 10.1177/08850666261489207

**Published:** 2026-09-19

**Authors:** Ramin Sam

**Affiliations:** 1 Department of Medicine, 36558 Zuckerberg San Francisco General Hospital, University of California, San Francisco, USA

## Authors Response:

To the Editor,

In response to Kocatas letter about the above study, a few point need to be pointed out. Here we had 503 episodes of severe lactic acidosis which is not a small number. The average AG-serum lactate concentration in this cohort was 6.9 ± 0.2 mEq/L which is well within the normal range of anion gap. Out of the 503 episodes only 171 had AG-serum lactate level of > 8.0, out of which 140 of them had either elevated serum albumin, serum phosphorus concentration, presence of ketones, presence β-hydroxybutyrate or a combination of the above. Out of these 140, 102 had elevated phosphorus levels which clearly can increase anion gap and very likely explained a lot of the increase in AG- serum lactate. An additional seven of these patients had AG- serum lacate – serum β-hydroxybutyrate level less than 8 mEq/L. Thus out of the 503 episodes, only 62 episodes (or 12.3%) did not have a really robust explanation for the elevated AG- serum lactate concentration. Additionally, another 31 episodes had either serum albumin concentration in higher range of normal according to our laboratory (normal range in our laboratory is defined between 3.4-4.8 g/dL), which can definitely explain an anion gap of let's say 8-10 mEq/L, presence of ketones in their urine or a combination of the above. If one excludes these patients also, then only 31/503 episodes or 6.2% of patients had an unexplained increase in AG -serum lactate levels. It would be logical to conclude that these patients likely suffered from another reason for increase in anion gap that was not diagnosed rather than assuming that AG – serum lactate concentration does not tell us about another cause for increase in serum anion gap. Even though this study has some limitations, we believe until better data is available, clinician should look for other causes of an elevation in serum anion gap, if the above delta is greater than 8.0 mEq/L. Not doing that would not make much sense.

Although we did include data within 24 h of serum lactate measurements, these were critically ill patients who had several metabolic panel measurements during the 24 h period. Thus, in the great majority of the episode, the values needed to calculate anion gap and lactate were measured well within a couple of hours of each other. To confirm the above, we looked at the first 20 patients and found that the lactate level was measured at the same time as the metabolic panel in 14/20 patients, 15 min from each other in 1, 0.5 h in 1, 1.5 h in 1, 2 h in another, 3 h 10 min in another and 9 h 15 min in the last patient. At this rate 70% of the values were obtained at the same time. The 24- h time limit was for values that were obtained much less often such as arterial blood gas, serum magnesium concentration, ketones and β-hydroxybutyrate levels.

The corrected anion gap and the strong ion difference were measured to more closely define the contribution of serum phosphorus, serum albumin, etc to the elevated anion gap. Unfortunately, these formulas don’t include the contribution of ketones, β-hydroxybutyrate and possibly other anions to the anion gap. However, with serum β-hydroxybutyrate measurement, it was clear that the increase in anion gap was explained by it in an additional seven patients.

As far as the inconsistencies in the report, the text of the article was written at a different time than Table 2 and we regret that we did not revise the text to reflect the correct number of patients. There were 171 episodes with AG-L > 8 mEq/L, 31 cases remained unexplained. The category count in Table 2 is correct but in addition there were 5 patient who had AG- L concentration of 8.0-8.1 and had mildly elevated serum phosphorus concentration of for example 5.8 mg/dL and/or serum albumin of around 4 g/dL which did not meet the criteria but in the opinion of the investigator likely did explain the AG – L level of ≥ 8 mEq/L.

At the end we would like to thank Dr Kocatas for pointing out issues of concern. However, we don’t believe the timing of the measurement is an issue with regard to AG – lactate measurement but it could be an issue in corrected anion gap or SIG measurements. We also strongly believe if one encounters a patient with elevated anion gap and serum lactate concentration but the AG – lactate level is > 10 for instance, one should look for another cause for anion gap metabolic acidosis.

